# A novel IL-10 signalling mechanism regulates TIMP-1 expression in human prostate tumour cells

**DOI:** 10.1038/sj.bjc.6600855

**Published:** 2003-05-13

**Authors:** M Wang, Y Hu, M E Stearns

**Affiliations:** 1 Department of Pathology and Laboratory Medicine, MCP-Hahnemann University, MS 435, 15th and Vine Sts., Philadelphia, PA 19102-1192, USA

**Keywords:** IL-10, normal prostate NPTX 1532 cells, TIMP-1, tyrosine phosphorylation

## Abstract

We have previously reported that interleukin 10 (IL-10) signalling stimulated activation of a specific enhancer element, termed HTE-1, to promote tissue inhibitor of matrix metalloproteinase1 (TIMP-1) expression in human bone metastatic PC-3 subclone (PC-3 ML) cells. Recently, we have identified an IL-10 responsive signal molecule, termed IL-10E1, which binds the HTE-1 element and cloned the gene encoding for the 22 kDa protein. In this paper, we have examined the mechanism of IL-10/IL-10 receptor signalling in two distinct human prostate cell lines, a ‘normal’ prostate epithelial cell line, termed NPTX-1532 and highly metastatic PC-3 ML tumour cells. Signalling cascade studies revealed that IL-10 stimulated tyrosine phosphorylation of JAK1 and TYK2 receptor kinases and tyrosine phosphorylation of IL-10E1. Phosphorylation, triggered IL-10E1's rapid translocation to the nucleus by 10–30 min. Deletion analysis combined with transient transfection experiments revealed that the n-terminal domain (∼74 a.a.) of the IL-10E1 protein, the nt-nls peptide, was stimulated by IL-10 to translocate to the nucleus and induce TIMP-1 expression. Site-directed mutagenesis further showed that phosphorylation of two tyrosine moieties (Y57 and Y62) of the nt-nls peptide was required for IL-10 activation of signalling and TIMP-1 expression. The data demonstrate, for the first time, that IL-10 receptor signalling of TIMP-1 expression is regulated by tyrosine phosphorylation of a novel gene, IL-10E1, in human prostate cells.

Changes in the relative levels and molar ratios of TIMPs and the MMPs are implicated in normal and pathological processes such as cancer, which involve connective tissue remodelling or degradation (Leco *et al*, 1992). Currently, it is thought that differential regulation of these genes might be achieved, in part, by various cytokines and growth factors, which allow responsive elements to either cooperate with or antagonise each other to modulate the expression of these genes (Leco *et al*, 1992). For example, there is a multiplicity of agents found to stimulate tissue inhibitor of matrix metalloproteinase1 (TIMP-1) expression in a variety of normal and malignant cell cultures. These range from serum, TNF *α*, bFGF, PDGF, EGF, phorbol esters, interleukin (IL)-1, IL-1 *β*, Il-6, and IL-11 (MacNaul *et al*, 1990; Campbell *et al*, 1991; Carcolo *et al*, 1991; Wright *et al*, 1991; Edwards *et al*, 1992; Roeb *et al*, 1993). In addition, inhibition of TIMP-1 expression occurs in several malignant cell lines in response to TGF *β*, all-*trans*-retinoic acid, female sex hormones, and dexamethasone (Campbell *et al*, 1991; Wright *et al*, 1991).

The regulation of TIMP-1 *vs* MMP expression may depend on specific receptor-mediated transactivating signals and/or promoter elements. For example, IL-6 stimulated TIMP-1 synthesis, but had no effect on MMP synthesis (Lacraz *et al*, 1993,^1995^); TGF *β* simultaneously increased TIMP-1 mRNA levels while decreasing MMP-2 mRNA content in fibroblasts (Overall *et al*, 1989); and steroids suppressed the expression of MMPs while simultaneously stimulating TIMP-1 production in uterine cervical fibroblasts (Sato *et al*, 1991). Similarly, interleukin-1 and TNF *α* were found to stimulate TIMP-1 production while up regulating MMP synthesis in cultured chondrocytes (Chua and Chua, 1990; Lotz and Guerne, 1991; Martel-Pelletier *et al*, 1991). However, in fibroblasts interleukin-1 and TNF *α* had little effect on TIMP-1 expression (MacNaul *et al*, 1990). The implication is that cell-type-specific signal cascades regulate gene expression.

Studies designed to identify determinant promoter regions and related transacting factors, which control TIMP-1 and MMP expression, would greatly aid in understanding the basis for differential regulation of these proteins in specific cell types. In this regard, we have examined the mechanisms regulating TIMP-1 expression in human prostate lines derived from nonmalignant and malignant tumours. Preliminary studies of the TIMP-1 promoter revealed that TIMP-1 expression was controlled, in part, by an 18 bp enhancer element (CACGATGACTCATCACTG), termed HTE-1, located in intron 1 upstream (−847 to −865 bp) of the 5′ ATG translational start site. Gel shift and chloramphenicol acetyl transferase (CAT) assays with promoter constructs demonstrated that the enhancer element binds a tentative regulatory protein(s), which was selectively expressed by nonmetastatic PC-3 subclones (i.e. 2 X N.I. PC-3 subclones which fail to metastasise when implanted in SCID mice) and not by the bone metastatic PC-3 subclone PC-3 ML human tumours (Wang *et al*, 1998). Moreover, and IL-10 independently stimulated a 7–9-fold increase in pCAT-HTE-1-driven CAT activity and TIMP-1 expression in transfected PC-3 ML cells. In addition, we found that IL-10, IL-4, and IL-6 differentially regulate TIMP-1, MMP-2, and MMP-9 mRNA and protein expression in HPV-18 immortalised human prostate cell lines derived from low and high Gleason score cancer tissue (Stearns *et al*, 1995). Western blot and Northern blot analysis clearly demonstrated that IL-10, IL-6, and IL-4 all upregulated TIMP-1 expression. In contrast, IL-10 and IL-4 (but not IL-6) downregulated MMP-2 mRNA and protein levels (Stearns *et al*, 1995). The data suggest that cytokine upregulation of TIMP-1 and coordinate downregulation of MMP-2 expression might control the molar ratio of TIMP-1 and MMP-2 to influence the level of protease activity and invasive behaviour of malignant prostate cancer cells.

In this paper, we have extended the initial studies to investigate the role of IL-10 receptor (IL-10R) signalling in the regulation of TIMP-1 expression in nonmalignant primary human prostate NPTX-1532 cell lines and malignant human prostate PC-3 ML cell lines. A novel IL-10 responsive signal molecule, termed IL-10E1, was identified which binds the HTE-1 element of the TIMP-1 promoter. We found that IL-10 triggered tyrosine phosphorylation of the IL-10R and the IL-10E1 protein to activate translocation of the IL-10E1 to the nucleus. Deletion analysis and site-directed mutagenesis showed that IL-10 specifically triggered tyrosine phosphorylation of tyrosines Y57 and Y62 to activate IL-10E1 signalling events and stimulate TIMP-1 expression. These data demonstrate for the first time that a novel IL-10 signal cascade regulates TIMP-1 expression in human prostate lines.

Note that the PC-3 ML and NPTX-1532 cells were selected for the signalling studies as both cell lines expressed the IL-10R, but only the PC-3 ML cells expressed detectable levels (i.e. in the 10–30 pg range) of the IL-10E1 protein. Thus, in the IL-10E1 transfection experiments, the translocation of the IL-10E1 protein (or truncated IL-10E1 fragments) to the nucleus could be examined in the presence or absence of endogenous IL-10E1.

## MATERIALS AND METHODS

### Cell cultures

We examined IL-10E1 expression in a variety of primary prostate cell lines. The NPTX-1532 and CPTX-1532 cells (generously provided by Drs Robert Bright and Susan Topalian, NIH-NCI, Bethesda, MD, USA) are normal prostate culture developed from normal prostate tissue extracted from human patients (Bright *et al*, 1997). These cells were immortalised with E6 and E7 transforming proteins of human papilloma virus serotype 16 and were maintained in Keratinocyte SFM (GIBCO BRL, Grand Island, NY, USA) containing 50 *μ*g ml^−1^ bovine pituitary extract, 5 ng ml^−1^ epidermal growth factor (EGF), and 5% foetal bovine serum (Biofluids, Rockville, MD, USA). Both MGEM and Keratinocyte SFM media contained 100 U ml^−1^ of penicillin G sodium and 100 *μ*g ml^−1^ streptomycin sulphate. Cells were routinely passaged by mild Trypsin–EDTA (GIBCO BRL, Grand Island, NY, USA) detachment and cultured in a humidified atmosphere of 95% air and 5% CO_2_ at 37°C. HPV-MLC7 cells (kindly provided by Dr Donna Peehl, Stanford University, CA, USA) are benign prostatic hyperplasia culture immortalised by human papilloma virus serotype 18 (Cohen *et al*, 1991). The HGPIN cell line was established in our laboratory from human prostate tissue showing high-grade prostatic intraepithelial neoplasia (HGPIN) histology. The HGPIN cultures were immortalised by HPV-18 transfection according to published methods (Wang *et al*, 1996). The BPH and HGPIN cell lines were maintained in Keratinocyte SFM (Gibco BRL, Grand Island, NY, USA) containing 5 ng ml^−1^ EGF, 50 *μ*g ml^−1^ bovine pituitary extract, and 5% foetal bovine serum (Biofluids, Rockville, MD, USA). All the different cell lines were transferred to their respective medium containing 10 nM DHT and 10 mM HEPES buffer, but without supplements (i.e. pituitary extracts and ITS) for 1 h prior to carrying out the IL-10 studies.

The 2 X N.I. PC-3 subclones and the bone metastasising PC-3 ML subclones derived from the parent human prostate PC-3 line were grown as previously described by our laboratory (Wang *et al*, 1998). In transfection experiments, the cell cultures were transiently transfected overnight and the cells exposed to IL-10 prior to harvesting and protein extraction from the cytoplasmic and nuclear compartments (Stearns *et al*, 1995). Unless stated otherwise, the cells were treated with IL-10 at 15 ng ml^−1^ for 1 h in all the experiments.

### Cloning the IL-10E1 gene

mRNA was isolated from PC-3 ML cells using a mRNA purification kit (PolyATtract system 1000′, Promega, Madison, WI). A phage vector ZAP-express vector (Stratagene, La Jolla, CA) was used to construct a cDNA library. Clones were screened three times according to Singh *et al* (1998) with a ^32^P-labeled oligonucleotide containing three repeats of the HTE-1 sequence. Positive phages were treated by *in vivo* excision to form a phagemid containing the cloned insert using a protocol described by the instruction manual of ZAP-cDNA Gigapack II Gold Cloning kit (Stratagene, La Jolla CA). The IL-10E1 cDNA was cloned in a GST-vector pGEX4T2 vector. The GST–IL-10E1 fusion protein was isolated using a glutathione Sepharose 4B beads, and IL-10E1 was purified utilising thrombin protease (27-0843-01) cleavage to separate IL-10E1 and GST proteins according to the methods of the manufacturer (Pharmacia Biotech, Piscataway, NJ, USA).

### Sequencing

DNA sequencing was done using ABI 377 and 373 A Stretch sequencers with Taq FS Dye Terminator or Dye Primer chemistry (DNA Sequencing Facility, University of Pennsylvania, USA). The protein sequence was deduced from the cDNA sequence using a GCG program. Peptide fragments generated from purified IL-10E1 protein were sequenced by the protein sequencing facility (University of Pennsylvania). Protein purity was verified by SDS–PAGE analysis.

### Electrophoretic mobility shift assays (EMSAs)

Cytoplasmic and nuclear proteins were prepared using published protocols (Wang *et al*, 1998). Crude extracts from recombinant phage lysogens were prepared as described by Singh *et al* (1998). The DNA–protein binding reaction and EMSA were carried out as described by Singh *et al* (1998). The DNA binding reaction mixture contained ∼100 000 c.p.m. of the ^32^P-end-labelled double-stranded oligonucleotide (∼1 ng), plus ∼5 *μ*g of crude protein extract, 1 *μ*g of poly (dI-dC)-poly (dI-dC) (Sigma) and buffer composed of 10 mM HEPES (pH 7.9), 50 mM NaCl, 1 mM DTT, 1 mM EDTA, and 5% glycerol. Samples were eluted on 8% SDS–PAGE gels and developed by autoradiography overnight.

### ELISAs

ELISAs (*A* 490 nm) were performed according to methods previously described (Stearns *et al*, 1995) using well-characterised polyclonal antibodies specific for TIMP-1 (Stearns *et al*, 1995). We used the TIMP-1 antibody at a dilution of 1 : 2000 (500 ng ml^−1^) and recorded the absorbance (*A* 490) for three different dilutions (∼5, 7.5, and 10 *μ*g ml^−1^ protein) of conditioned medium from NPTX-1532 cells. Cells were plated on human collagen IV (100 *μ*g well^−1^ in 12 well dishes) at 1 × 10^6^ cells ml^−1^ for 24 h and exposed to IL-10 for 24 h prior to collecting the conditioned medium. The measurements were normalised to 10 *μ*g ml^−1^ protein and the total amounts of antigen ml^−1^ determined from an ELISA standard curve (Stearns *et al*, 1995). Protein levels were measured according to the methods of Bradford (1976).

### Site-directed mutagenesis

Site-directed mutagenesis and deletion analysis were carried out according to the methods described by Sambrook *et al* (1989). The deleted or mutated fragments were generated from the IL-10E1 cDNA using specific primers and PCR (∼35 cycles amplification). The fragments were then subcloned and sequenced (DNA Sequencing Facility, University of Pennsylvania) to confirm the specific deletions and mutations.

### Subcloning the IL-10E1 fragments

To generate the different IL-10E1 subclones, ZAP-cDNA synthesis, ligation of the ZAP-cDNA into the Uni-ZAP XR vector arms, and packaging of recombinant phage DNA were performed using a lambda phage vector-ZAP Express cDNA vector and protocol of the ZAP-cDNA Gigapack^R^ II Gold Cloning Kit (Stratagene Inc., San Diego, CA, USA). The cDNA fragments cloned encoded for the following peptide fragments of the IL-10E1 gene, including:


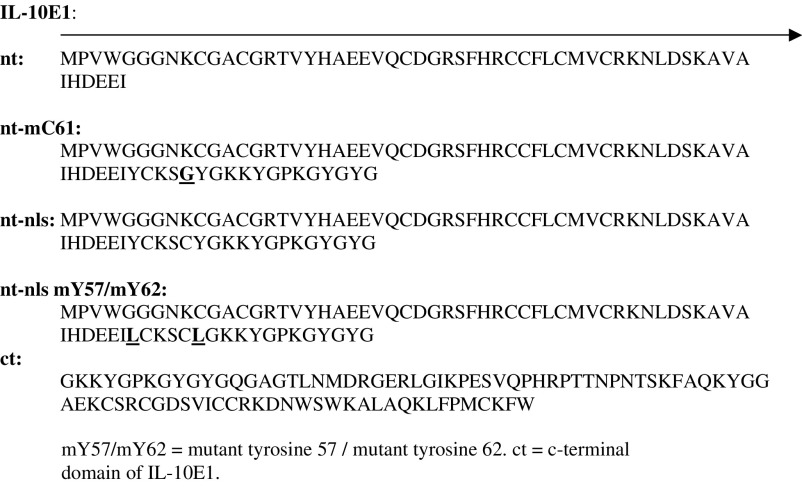



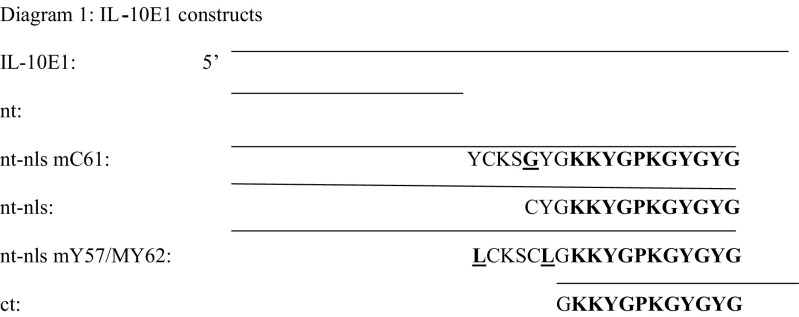


Amino acids bolded and underlined were mutated. The nuclear localisation signal (nls) was bolded.

Mc 61=mutant tyrosine 61.

### Transient transfection experiments

NPTX-1532 or PC-3 ML cells were transfected overnight with 4 *μ*g of recombinant phagemid using 8 *μ*g of Lipofectin reagent according to protocol described by the manufacturer (Gibco Life Technologies Inc., Boston, MA, USA). Western blots were according to the methods of Towbin *et al* (1979).

### Source of reagents

The following indicate the reagents (source): IL-10 (Schering Corp., Kenilworth, NJ, USA); IL-10 and IL-10 receptor antibodies (Schering Plough; Kevin Moore, DNAX Inc., San Diego, CA, USA); (Liu *et al*, 1994); rabbit antibodies against human JAK1, JAK2, TYK2 (UBI, Inc. Saranac Lake, NY, USA); Protein A–Sepharose and Protein-G Sepharose beads (Pharmacia, Piscataway, NJ, USA); PVDF membranes (Immobilon-P, Millipore, Bedford, MA, USA). Antiphosphotyrosine antibody PY20 (Sigma Sci., St Louis, MO, USA).

## RESULTS

### Cloning of IL-10E1 gene

**Figure 1:**
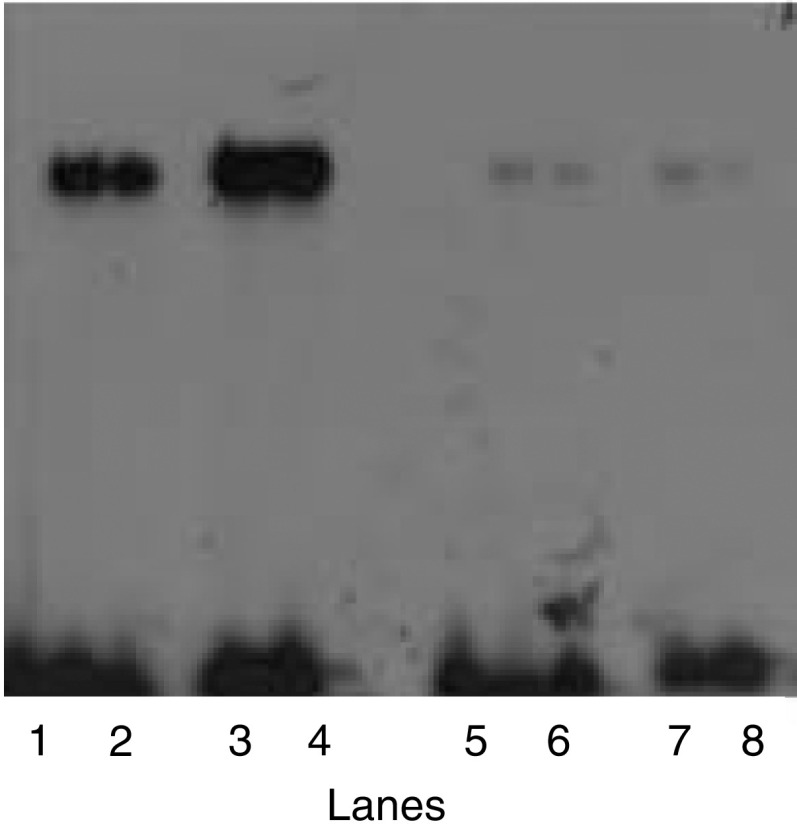
Electrophoretic mobility shift assays (EMSAs) showing that the ^32^P-end-labelled double-stranded HTE-1 oligonucleotide (∼1 ng, ∼100 000 c.p.m.) binds rIL-10E1 protein and native nIL-10E1 protein (5 *μ*g/lane^−1^) from PC-3 ML cells. The probe was incubated with protein (5 *μ*g) from (lanes 1–2) two rIL-10E1 phagemid clones; (lane 3) PC-3 ML cells (nIL-10E1); (lane 4) rIL-10E1-transfected NPTX-1532 cells; (lane 5) a phagemid clone and (lane 6) a bacteria culture. In control experiments (lane 7), the nuclear protein extracts were obtained from PC-3 ML cells exposed to IL-10 in the presence of IL-10R antibodies (1 : 100 dilution); or (lane 8) from IL-10-treated cells where the nuclear protein extracts were preincubated (for 30 min) with excess ‘cold-HTE-1 oligonucleotide’ at concentrations of 10 ng ml^−1^, which competitively blocked labelled oligonucleotide binding to rIL-10E1 protein.

An IL-10 responsive signal protein, termed IL-10E1, was cloned from a phage expression cDNA library utilising a triplicate repeat of the HTE-1 sequence, an enhancer element of the TIMP-1 promoter (Wang *et al*, 1998). In preliminary studies, EMSAs showed that a protein present in lysogen extracts from rIL-10E1 recombinant phagemid clones binds the HTE-1 oligonucleotide (Figure 1

, lanes 1–2). The band shift mobility was identical to that observed with the native protein isolated from human prostate PC-3 ML cells (Figure 1, lane 3) and rIL-10E1 protein extracted from rIL-10E1 transfected NPTX-1532 cells (Figure 1, lane 4). In comparison, protein from a randomly selected phagemid clones (Figure 1, lane 5) and the bacteria extract alone (Figure 1, lane 6) failed to bind the HTE-1 probe. In control experiments, the nuclear protein extracts were obtained from PC-3 ML cells exposed to IL-10 in the presence of IL-10R antibodies (1 : 100 dilution) (Figure 1, lane 7), or the protein extracts were preincubated with excess ‘cold HTE-1 oligonucleotide’ at concentrations ranging from 10 ng ml^−1^ for 30 min (Figure 1, lane 8). Note that a total of five separate rIL-10E1 phagemid clones derived from three different cDNA libraries yielded a recombinant protein that bound the HTE-1 probe.

### Sequencing

Sequencing of three independently derived IL-10E1 clones revealed that the IL-10E1 gene was ∼95% homologous with CSRP-2 and hsmLIM domain genes (Weiskirchen *et al*, 1997).

**Figure 2:**
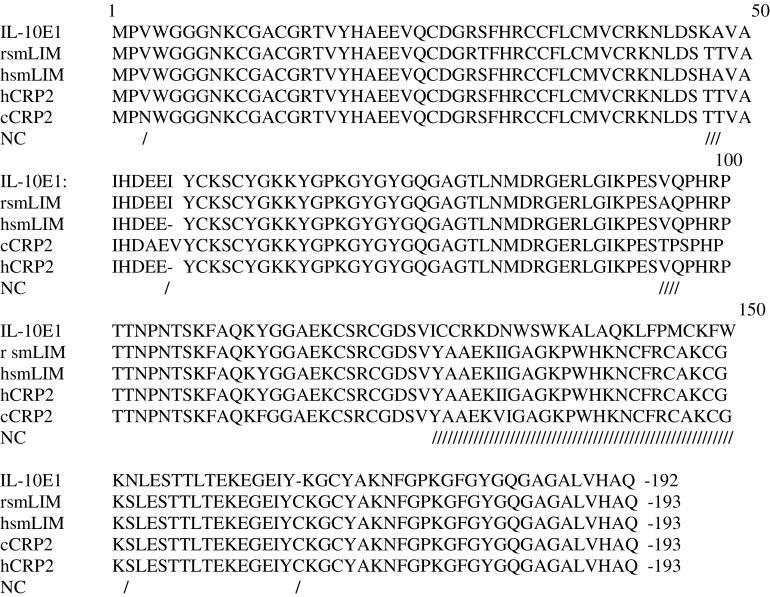
Amino-acid sequence of IL-10E1 compared with amino-acid sequences of several other genes with 90–95% homology. Deduced amino-acid sequences for IL-10E1; smooth muscle LIM (rsmLIM); human smooth muscle LIM (hsmLIM); chick CRP-2 (cCRP2); and human CRP-2 (hCRP2); NC-non-concensus amino acids.

IL-10E1's homology with CSRP-2 and hsmLIM was reduced to ∼90% in a region spanning exons 2 and 3 (i.e. ∼+451 to +490 bp) (Weiskirchen *et al*, 1997). The deduced amino-acid sequences revealed that the IL-10E1 protein had an open reading frame of 192 amino acids (a.a.) (∼22 kDa) (Figure 2

). A classical LIM-domain sequence (CX2C17HX2CX2C17CX2C) was detected in the n-terminal region with a GY-rich box tail (bolded) and a nuclear localisation signal-(underlined) (**YCKSCYG**KKYGPKGYGY).

### Western blot studies

**Figure 3:**
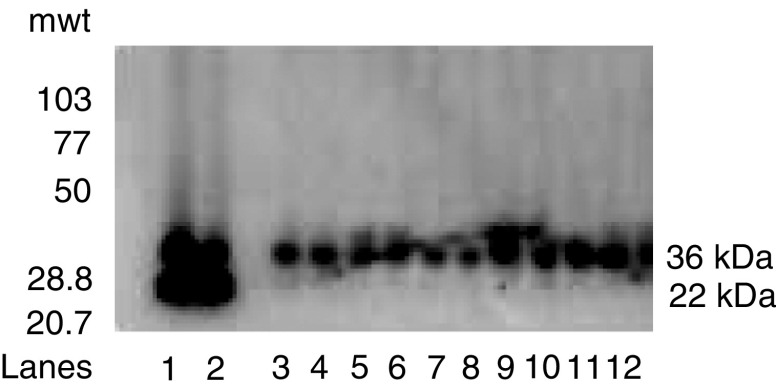
Western blots with IL-10E1 antibodies of whole-cell extracts from (lanes 1–2) PC-3 ML cells and CPTX-1532 cells, respectively; (lane 3–12) four BPH lines, two HGPIN lines and four normal NPTX-1532 cell lines, respectively, are shown. Cells were lysed, and the protein (1 mg ml^−1^) was electrophoresed on SDS–PAGE and Western blotted with a mixture of IL-10E1 (0.1 *μ*g ml^−1^) and GADPH (0.5 *μ*g ml^−1^) antibodies. Top band (36 kDa) GADPH; bottom band (22 kDa) IL-10E1.

**Figure 4:**
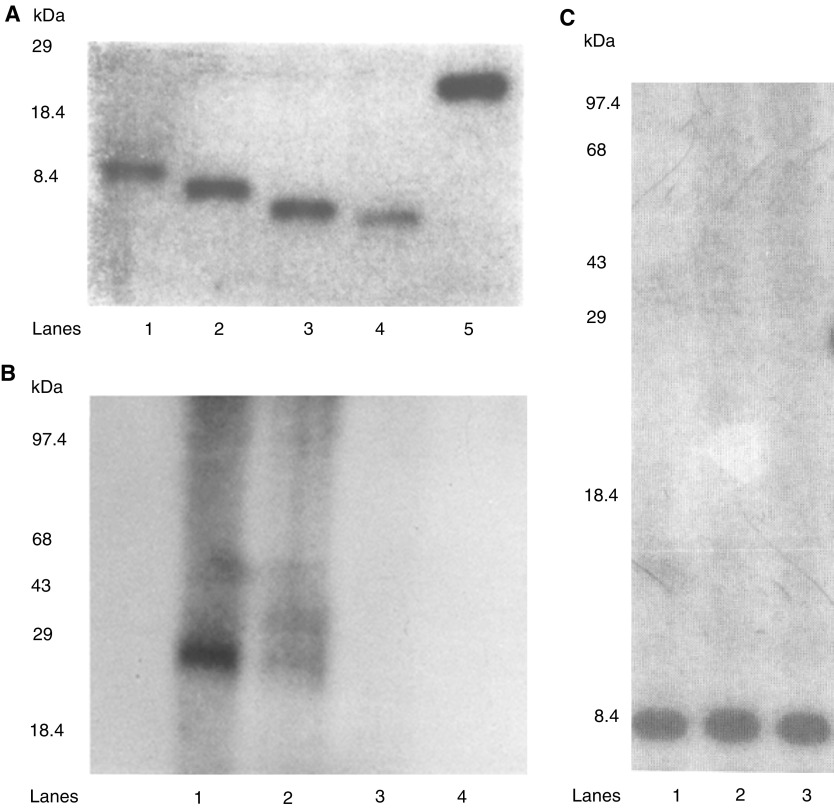
(**A**) Western blots (of the same gel) with IL-10E1 antibodies (8–15% SDS–PAGE) of recombinant peptide fragments of the IL-10E1 gene, including: (lanes 1–4) rnt-nls, rnt-nls mC61, rnt-nls mY57/mY62, and rnt-nls mY57/Y62, respectively and (lane 5) recombinant IL-10E1. (**B**) Western blots with IL-10E1 antibodies (8% SDS–PAGE) of recombinant IL-10E1 peptides, including: (lanes 1–2) rnt-nls; (lane 3) rct and (lane 4) rnt fragments. (lane 2) the IL-10E1 antibodies were preabsorbed with excess rIL-10E1 antigen. (**C**) Western blots of the same gel with IL-10E1 antibodies (8% SDS–PAGE) of crude cytoplasmic protein extracts from NPTX-1532 cells transfected overnight with recombinant (lane 1) rnt-nls; (lane 2) rnt-nls mC61; (lane 3) nt-nls mY57/mY62 constructs.

Rabbit polyclonal antibodies were generated against a 22 a.a. peptide (YCKSCYGKKYGPKGYGYGQGAG) located near the n-terminal domain of the IL-10E1 protein (see diagram 1 in Materials and Methods and Figure 2). Western blot analyses on whole-cell extracts demonstrated that IL-10E1 was expressed at roughly equivalent levels in PC-3 ML and CPTX-1532 cells (Figure 3

, lanes 1–2), but was not expressed in the four different BPH, two HGPIN and the four different NPTX-1532 cell lines (Figure 3, lanes 3–12, respectively). Note that CPTX-1532 cells are a malignant primary prostate line derived from the same prostate as the normal NPTX-1532 cells. Western blots further showed that the IL-10E1 antibodies specifically recognised the rIL-10E1 protein (22 kDa, Figure 4A

, lane 5).

Truncated peptide fragments of the IL-10E1 protein were produced for studies of the role IL-10E1 in IL-10R signalling (see Diagram 1). Western blots also showed that the IL-10E1 antibodies recognised peptide fragments of the IL-10E1 protein, which, were isolated from recombinant phagemid clones expressing truncated rnt-nls; rnt-nls mC61, and rnt-nls mY57/mY62 peptides (Figure 4A, lanes 1–4, respectively). Note that the samples were eluted on a 8–15% gradient gel and the peptide fragments appear to vary in size slightly as a result of one or two amino-acid differences in the amino-acid sequence (Figure 4A). Interestingly, the IL-10E1 antibodies recognised the rnt-nls-peptides (Figure 4B, lane 1) and binding was specifically competed by preabsorbing the antibody with rIL-10E1 protein (Figure 4B, lane 2). However, the antibodies failed to bind rct and rnt peptides, respectively (Figure 4B, lanes 3–4), which were generated from the c-terminal domain of the IL-10E1 protein and missing the ‘**YCKSCYG**KKYGPKGYGY’ region of the rnt-nls peptide.

Western blotting studies further showed that following transfection of NPTX-1532 cells with either rnt-nls, rnt-nls mC61, or rnt-nls mY57/mY62 constructs (Figure 4C, lanes 1–3), respectively, the truncated peptides and the intact protein were expressed in crude cytoplasmic extracts of the cells.

### IL-10 signalling studies

**Figure 5:**
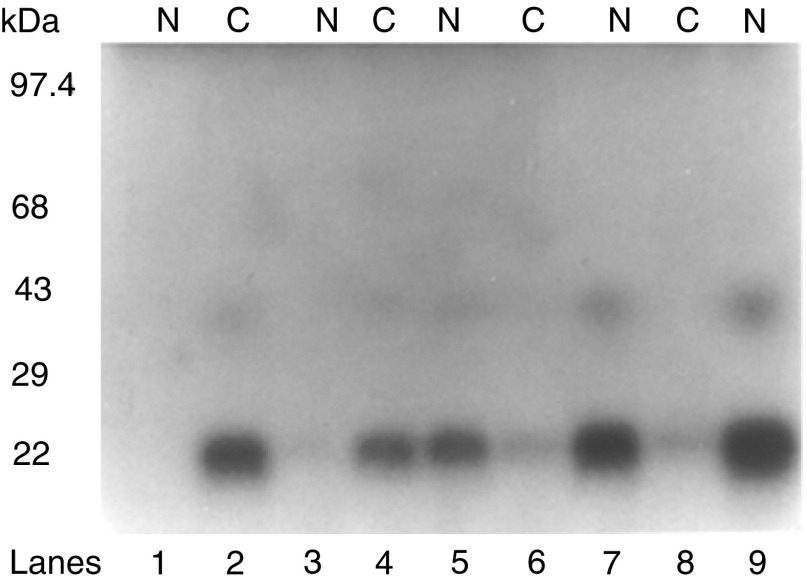
Western blots (8% SDS–PAGE) with IL-10E1 antibodies comparing (matching) cytoplasmic (C) (lanes 2, 4, 6, 8) and nuclear (N) (lanes 1, 3, 5, 7, 9) protein extracts from NPTX 1532 cells transfected with the recombinant rIL-10E1 construct. Prior to harvesting, cells were exposed to IL-10 for: (lanes 1–2) 0; (lane 3–4) 10; (lane 5–6) 30; (lane 7–8) 60 and (lane 9) 120 min.

**Figure 6:**
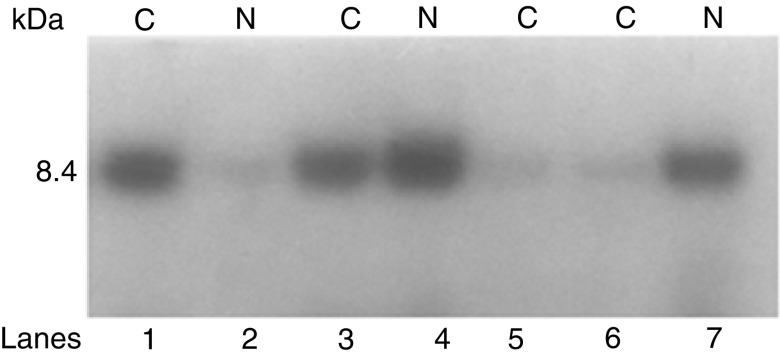
Western blots (8% SDS–PAGE) with IL-10E1 antibodies comparing (lanes 1, 3, 5, 6) cytoplasmic (C) and (2, 4, 7) nuclear protein extracts from NPTX 1532 cells transfected with the recombinant rnt-nls construct. Prior to harvesting, cells were exposed to IL-10 for: (lanes 1–2) 0; (lanes 3–4) 10; and (lanes 5–7) 30 min.

These studies were initially carried out in a normal NPTX-1532 prostate cell line, which expressed barely detectable levels of TIMP-1 and which failed to express endogenous IL-10 E1. Following transient transfection of the NPTX-1532 cells with the rIL-10E1 construct, cells were treated with IL-10 (15 ng ml^−1^ for 0–120 min). Western blots of nuclear and cytoplasmic protein extracts revealed that rIL-10E1 was present in cytoplasmic extracts at time zero and after 10 min stimulation with IL-10 (Figure 5

, lanes 2, 4, respectively). rIL-10E1 was not detected in the nuclear extracts from these cells (Figure 5, lanes 1, 3). After 30, 60, and 120 min stimulation, the majority of the rIL-10E1 was now present in the nuclear extracts (Figure 5, lanes 5, 7, 9, respectively). The IL-10E1 protein was no longer detectable in the cytoplasmic extracts by ∼30–60 min exposure to IL-10 (Figure 5, lanes 6 and 8) or by 90 min (not shown). Similar studies of NPTX-1532 cells transfected with the truncated rnt-nls construct showed that the ∼8.4 kDa peptide was expressed in the cytoplasmic fractions in unstimulated cells (Figure 6

, lane 1) and not in the nuclear fractions (Figure 6, lane 2). Following IL-10 treatment for 10 min, rnt-nls was detected in both cytoplasmic and nuclear protein extracts (Figure 6, compare lanes 3, 4). In comparison, by 30 min stimulation rnt-nls was no longer detected in cytoplasmic extracts (Figure 6, lanes 5–6), but was detected in the nuclear extracts (Figure 6, lane 7).

**Figure 7:**
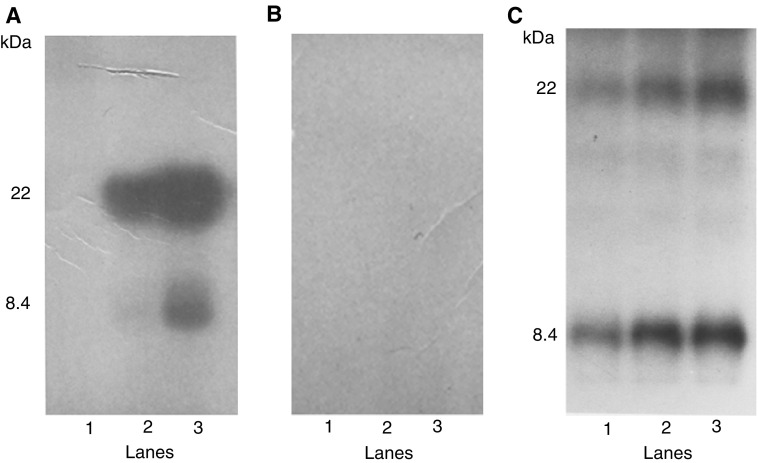
(A–C) Western blots (6% SDS–PAGE) with IL-10E1 antibodies comparing nuclear protein extracts from PC-3 ML cells transfected with a recombinant rnt-nls construct and exposed to: (**A**) IL-10 and (**B**) IL-10 plus IL-10R antibodies for (lane 1) 10; (lane 2) 30; and (lane 3) 60 min. (**C**) Lanes 1–3 show blots of the matching cytoplasmic fractions from the cells in Figure (B), lanes 1–3, respectively. (lower band-rnt-nls); (upper band-nIL-10E1).

Identical studies were also carried out with PC-3 ML human prostate tumour cells, which normally expressed endogenous IL-10E1 protein. Following transfection with the rnt-nls construct, both nIL-10E1 and rnt-nls were expressed in cytoplasmic extracts (data not shown). Following IL-10 stimulation, the presence of both native nIL-10E1 and the rnt-nls peptide was assayed in nuclear protein extracts. Neither protein was detected in the nuclear extracts after 10 min stimulation (Figure 7A

, lane 1). However, both the intact nIL-10E1 protein and the nt-nls peptide were detected in the nuclear protein fractions after ∼30–60 min (Figure 7A, lanes 2–3). Control experiments in Figure 7B (lanes 1–3) showed that in the presence of IL-10R antibodies, IL-10 failed to stimulate nIL-10E1 and rnt-nls translocation to the nucleus. Both the 22 kDa nIL-10E1 protein and 8.4 kDa rnt-nls peptide remained in the cytoplasmic fractions of cells exposed to IL-10 in the presence of IL-10R antibodies for 10, 30, and 60 min (Figure 7C, lanes 1–3). The data indicate that the IL-10/IL-10R axis regulates IL-10E1 signalling in nonmalignant and malignant prostate lines and indicates that the nt-nls peptide may, in part, be functionally equivalent to the intact nIL-10E1 protein.

### JAK/TYK kinase studies

**Figure 8:**
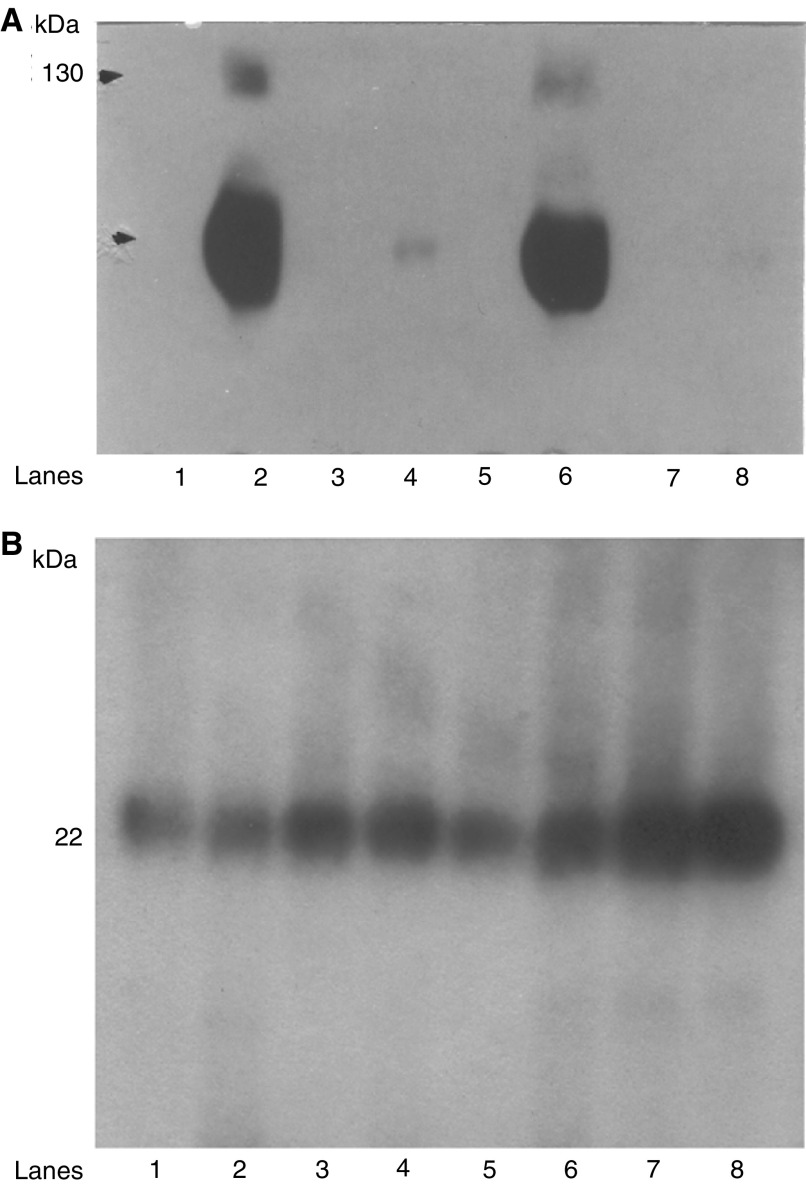
(**A**) Western blots (8% SDS–PAGE) with: Fig. 8A: (lanes 1, 2) JAK 1, (lanes 3, 4) secondary antibody alone, (lanes 5, 6) TYK2 and (lanes 7, 8) JAK 3 antibodies. NPTX 1532 cells were transfected with recombinant rnt-nls and treated with IL-10 in the (lanes 1, 3, 5, 7) presence and (lanes 2, 4, 6, 8) absence of IL-10 receptor antibodies (1 : 100 dilution). Phosphorylated proteins were immunoprecipitated with PY20 antibodies from whole-cell extracts for Western blots. (arrowhead) Top band-specific antibody binding (double arrowhead) Lower band-nonspecific antibody binding. (**B**) Lanes 1–8. Western blots with IL-10E1 antibodies of whole-cell extracts from IL-10E1-transfected NPTX-1532 cells. IL-10E1 was uniformly expressed in the different studies described in (A), Lanes 1–8.

We postulated that the IL-10 receptor might regulate the IL-10E1 signalling cascade. To test this possibility, we initially employed NPTX-1532 cells transfected with the rIL-10E1 construct. Cells were treated with IL-10 and harvested to prepare crude protein extracts for immunoprecipitation assays with excess phosphotyrosine-specific antibodies (PY20) (Figure 8A

). The immunoprecipitates were then electrophoresed on SDS–PAGE gels and blotted with JAK1- and TYK2-specific antibodies. Both antibodies blotted a ∼130 kDa band on the gels that corresponded to the JAK1 and TYK2 proteins (arrowhead, Figure 8A, lanes 2 and 6, respectively). In comparison, JAK3 antibodies failed to blot any detectable protein (Figure 8A, lane 8), indicating that an IL-10-specific receptor was involved. Western blot studies further showed that in cells exposed to IL-10 in the presence of IL-10R antibodies, the PY20 antibodies failed to immunoprecipitate JAK1, TYK2, or JAK3 (Figure 8A, lanes 1, 5, 7, respectively) from crude cell extracts, presumably as a result of the receptor antibodies blocking IL-10's induction of IL-10 receptor phosphorylation. Figure 8A, lanes 3 and 4 show that secondary antibodies alone failed to blot the 130 kDa JAK and TYK bands. Note that the lower band (double arrowhead) in lanes 2 and 6 appeared to be a nonspecific band inexplicably blotted by both JAK1 and TYK2 antibodies. Western blots with IL-10E1 antibodies (Figure 8B, lanes 1–8) confirmed that 22 kDa rIL-10E1 protein was uniformly expressed in the preparations blotted in Figure 8A, and indicated that differences in protein content were not a limiting factor in Figure 8A.

**Figure 9:**
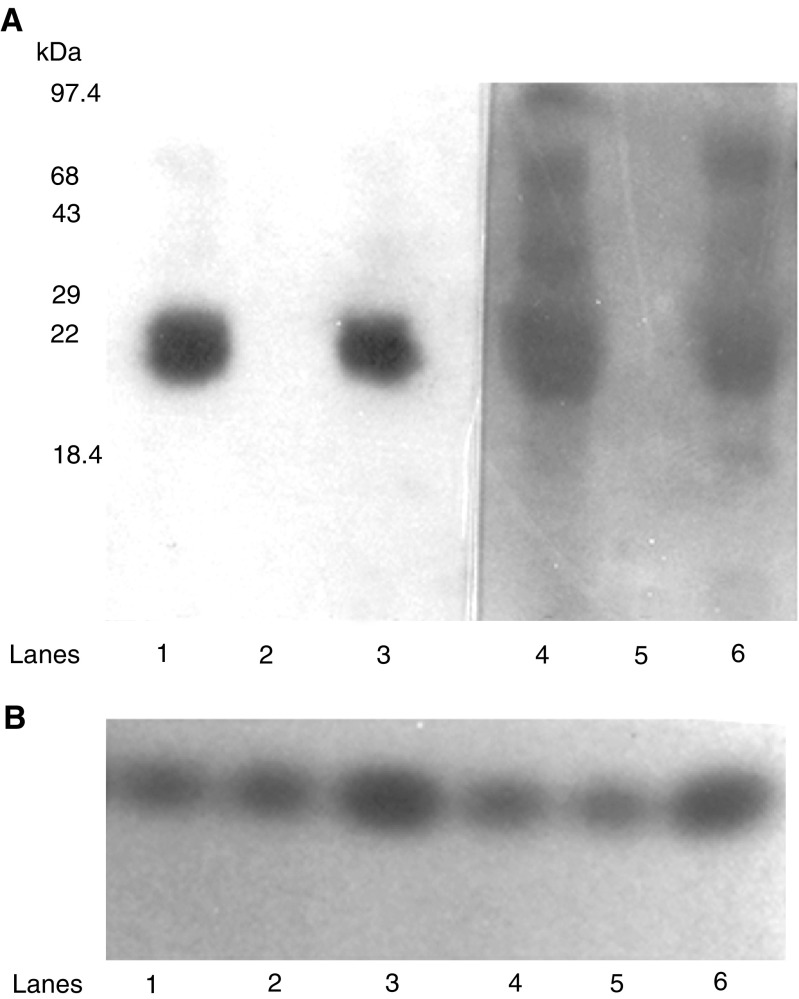
(**A**) Western blots with (lanes 1–3) IL-10E1 and (lanes 4–6) PY20 antibodies of immunoprecipitates obtained from nuclear protein extracts of rIL-10E1-transfected NPTX-1532. Cells were pretreated for 60 min with: (lanes 1 and 4) IL-10; (lanes 2 and 5) IL-10+IL-10R antibodies; and (lanes 3 and 6) IL-10 plus secondary IgG antibodies. (**B**) Lanes 1–6. Western blots with IL-10E1 antibodies of whole-cell extracts from untreated NPTX-1532 cells in Figure 8A, lanes 1–6, respectively.

The immunoprecipitation studies were extended to examine whether rIL-10E1 protein in transfected NPTX-1532 cells was also phosphorylated in response to IL-10 ligand. Following IL-10 treatment for 60 min, immunoprecipitates were isolated with PY20 antibodies from nuclear protein extracts. Western blots with IL-10 E1 antibodies showed that the immunoprecipitates from IL-10-treated cells contained IL-10E1 protein (Figure 9A

, lanes 1, 3). In addition, immunoprecipitates prepared with IL-10E1 antibodies were blotted with PY20 antibodies which labelled a 22 kDa protein (Figure 9A, lanes 4, 6), plus 1–2 associated proteins of unknown composition. In control studies, IL-10 in the presence of IL-10R antibodies failed to induce rIL-10E1 translocation to the nucleus (Figure 9A, lanes 2, 5). That is, both PY20 and IL-10E1 antibodies failed to immunoprecipitate the 22 kDa protein from nuclear extracts of these cells, and Western blots with IL-10E1 (Figure 9A, lane 2) and PY20 (Figure 9A, lane 5) antibodies failed to detect the 22 kDa protein in the respective immunoprecipitates. Control experiments where the cells were incubated with IL-10 plus secondary IgG antibody showed that the secondary antibodies did not block IL-10 activation of the IL-10R. In these latter assays, the IL-10E1 protein was immunoprecipitated with both PY20 (Figure 9A, lane 3) and IL-10E1 (Figure 9A, lane 6) antibodies and blotted with IL-10E1 and PY20 antibodies, respectively. Western blots of whole-cell extracts of the rIL-10E1-transfected cells confirmed that rIL-10E1 protein was uniformly expressed by the NPTX-1532 cells in these experiments (Figure 9B, lanes 1–6).

### Site-directed mutagenesis studies

**Figure 10:**
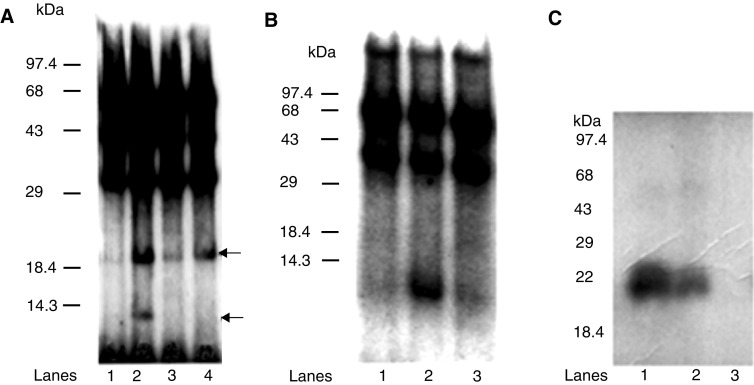
(**A**) Immunoprecipitates with PY20 antibodies of nuclear extracts from PC-3 ML cells. Cells were transiently transfected o.n. with recombinant (lanes 1–2) rnt-nls (∼8.4 kDa); and (lane 3–4) rnt-nls mY57/MY62 constructs and then (lanes 1, 3) untreated; or (lanes 2–4) treated with IL-10. Arrowhead: nIL-10E1–22 kDa; double arrowhead: 8.4 kDa peptide. The three bands at the top of the gel are phosphorylated proteins present in all the PY20 immunoprecipitates. (**B**) Immunoprecipitates with PY20 antibodies of nuclear extracts from NPTX-1532 cells. Cells were transfected with (lane 1) rnt-mY57/mY62 and (lanes 2–3) rnt-nls constructs. The cells were exposed to IL-10 for (lane 1) 0 min and (lane 2) 60 min, and (lane 3) IL-10 + IL-10R abs for 60 min. (**C**) Western blots with IL-10E1 antibodies of cytoplasmic protein extracts from the NPTX-1532 cells confirmed that (lane 1) rnt-nls, (lane 2) rnt-mY57/mY62 peptides were expressed in the cytoplasm of untreated cells. (Lane 3) cells transfected with vector alone.

In attempts to identify whether specific tyrosine groups were phosphorylated in response to IL-10 ligand, PC-3 ML cells were transfected with either rnt-nls (Figure 10A

, lanes 1–2) or mutant rnt-nls mY57/mY62 constructs (Figure 10A, lanes 3–4). In rnt-nls-transfected cells, gel autoradiography of immunoprecipitates isolated with the PY 20 antibodies showed that the nuclear protein extracts from untreated cells did not contain either nIL-10E1 or rnt-nls (Figure 10A, lane 1). Following IL-10 treatment, the nuclear extracts contained both nIL-10E1 (arrowhead) and rnt-nls peptide (double arrowhead) (Figure 10A, lane 2). In PC-3 ML cells transfected with the rnt-nls mY57/mY62 expression vector, PY20 antibodies failed to immunoprecipitate the nt-nls mY57/mY62 peptide from nuclear extracts of untreated (Figure 10A, lane 3) and IL-10-treated cells (Figure 10A, lane 4). In comparison, the nIL-10E1 protein was barely detectable in nuclear extracts of untreated cells (Figure 10A, lane 3), but was significantly elevated in nuclear extracts of IL-10-treated cells (Figure 10A, lane 4, arrowhead). Western blots with IL-10E1 antibodies of cytoplasmic extracts verified that nIL-10E1 and rnt-nls mY57/mY62 peptides were both expressed in the cytoplasmic protein extracts of untreated PC-3 ML cells (data not shown). Western blots with IL-10E1 antibodies further showed that the immunoprecipitates prepared with PY20 antibodies from cytoplasmic and nuclear protein fractions of the IL-10-treated cells did not contain detectable levels of rnt-nls mY57/mY62 peptide, indicating that the PY20 antibodies failed to recognise nonphosphorylated protein. The nIL-10E1 protein was detected in Western blots of the PY20 antibody immunoprecipitates prepared from the nuclear extracts of IL-10-treated cells. The truncated rnt-nls mY57/mY62 peptide was not detected in these assays (data not shown), presumably because IL-10 failed to induce its translocation to the nucleus.

Identical experiments were carried out with normal NPTX-1532 cells transfected with either rnt-nls or rnt-nls mY57/mY62 constructs (Figure 10B). Gel autoradiography of immunoprecipitates prepared with PY20 antibodies showed that nuclear extracts from cells exposed to IL-10 for 120 min (i.e. NPTX-1532 cells transfected with the rnt-nls mY57/mY62 construct) did not contain the rnt-nls mY57/mY62 peptide (Figure 10B, lane 1). In contrast, the rnt-nls peptide was detected in nuclear extracts (i.e. from NPTX-1532 cells transfected with the rnt-nls construct) following exposure of the cells to IL-10 for 120 min (Figure 10B, lane 2). That is, the phosphorylated rnt-nls peptide was immunoprecipitated with PY20 antibodies from the nuclear extracts of these cells (Figure 10B, lane 3). Control experiments showed that the rnt-nls peptide was not immunoprecipitated from nuclear extracts of NPTX-1532 cells (transfected with the rnt-nls construct) exposed to IL-10 in the presence of IL-10R antibodies (Figure 10B, lane 3). Western blots with IL-10E1 antibodies of cytoplasmic extracts from the untreated NPTX-1532 cells confirmed that both rnt-nls (Figure 10C, lane 1) and rnt-mY57/mY62 peptides (Figure 10C, lane 2) were expressed in the NPTX-1532 cells lines transfected with their respective constructs. The rnt-nls peptide was not detected in NPTX-1532 cells transfected with vector alone (Figure 10C, lane 3). These data indicate that an intact rnt-nls peptide (i.e. with Y57 and Y62 tyrosines) was required for the nuclear localisation of the rnt-nls peptide and the IL-10E1. Note that the 2–3 bands at the top of the gel in Figure 10A and B were phosphorylated proteins of unknown identity, which were uniformly precipitated with the PY20 antibodies from extracts of both untreated and IL-10-treated cells.

### Regulation of TIMP-1 expression

**Figure 11:**
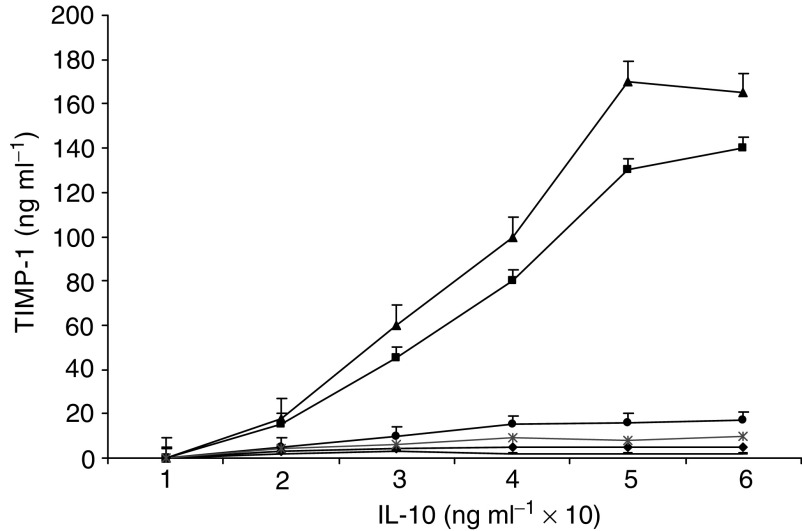
ELISA measurements of TIMP-1 secreted over 24 h by NPTX-1532 cells in the presence of IL-10 at concentrations ranging from 10 to 60 ng ml^−1^ for 24 h. NPTX-1532 cells were (−) not transfected; (x) mock-transfected with vector alone; or transfected with (▴) rIL-10E1; (▪) rnt-nls; and (⧫) rnt-mY57/mY62 expression vectors. NPTX-1532 cells were transfected with (•) rnt-nls and exposed to IL-10 in the presence of IL-10R antibodies. Values represent the mean+1 s.d. from at least three experiments and triplicate measurements per experiment.

Preliminary studies were carried out to assess if IL-10 activation of rIL-10E1 or the rnt-nls peptide induced TIMP-1 expression in transfected NPTX-1532 cells. ELISA measurements showed that NPTX-1532 cells (Figure 11

, -) and mock-transfected NTPX-1532 cells (Figure 11, **X**) (i.e. cells transfected with vector alone) normally expressed (secreted) barely detectable levels of TIMP-1 protein in the absence or presence of increased levels of IL-10. However, following transient transfection overnight with either the IL-10E1 or the nt-nls construct, IL-10 (0–60 ng ml^−1^ for 24 h) induced TIMP-1 expression in a dosage-dependent manner in these NPTX-1532 subclones (Figure 11, ▴▪). IL-10 receptor antibodies blocked IL-10 induction of TIMP-1 expression by cells transfected with the rnt-nls construct (Figure 11, •) and the rIL-10E1 expression vector (data not shown). In comparison, NPTX-1532 subclones transfected with the rnt-mY57/mY62 construct failed to secrete TIMP-1 in response to IL-10 at increased dosages for 24 h (Figure 11, ⧫). These experiments indicate that IL-10 activation of the rIL-10E1 protein or the truncated rnt-nls peptide stimulated TIMP-1 production. Since IL-10 failed to induce TIMP-1 expression in NPTX-1532 cells transfected with the nt-mY57/mT 62 peptide, we suggest that the intact nt-nls peptide was required for the signalling cascade and that IL-10-induced phosphorylation of specific tyrosine groups (e.g. Y57 and Y62) may regulate activation of the IL-10E1 protein and nt-nls peptide to control TIMP-1 synthesis.

## DISCUSSION

In two earlier papers, we found that the IL-10 stimulated activation of the HTE-1 enhancer element of the TIMP-1 gene to activate TIMP-1 expression in malignant human prostate cell lines (Wang *et al*, 1998,^2002^). In this paper, the data significantly extended these studies and showed that IL-10 ligand binding to the IL-10 receptor activated a signal cascade, which rapidly activated TIMP-1 expression in normal and malignant prostate cell lines. That is, IL-10 binding to the IL-10R induced tyrosine phosphorylation of the JAK 1/TYK2 kinases, tyrosine phosphorylation of Y57 and Y62 tyrosines in the ‘n-terminal’ domain of the IL-10E1 protein, and the rapid transport to the nucleus of the IL-10E1 protein. Immunoprecipitation assays showed that phosphorylation of Y57/Y62 tyrosines triggered redistribution of the intact IL-10E1 protein and the nt-nls fragment in both normal and malignant human prostate cell lines. The nt-nls mY57/mY62 fragments containing mutated tyrosines were not phosphorylated following IL-10 treatment of transfected cells and the nt-nls peptide failed to migrate to the nucleus in these cells. In addition, nuclear translocation of the nt-nls fragment (and presumably the intact IL-10E1 protein as well) required the nls, but not the remaining c-terminal domain of the IL-10E1 protein. This indicates that the mechanism of activation of the nls region may involve changes in protein–protein interaction, or perhaps conformational changes in the IL-10E1 protein mediated by tyrosine phosphorylation of Y57 and Y62 groups. In sum, the data from the site-directed mutagenesis and deletion analysis studies uniformly confirmed that the n-terminal domain, and specifically the region spanning about 17 a.a.'s (e.g. YCKSCYG**KKYGPKGYGY**), were primarily responsible for nuclear translocation of the IL-10E1 protein. We believe that this domain may possibly control binding to the HTE-1 element and activation of TIMP-1 expression. The data presented in Figure 10 clearly showed that IL-10 activation of IL-10E1 signalling serves to upregulate TIMP-1 in NPTX-1532 cells, but the precise mechanism controlling the molecular process has yet to be defined. Preliminary EMSA studies in our laboratory have shown that the nt-nls fragment binds the HTE-1 site, but the truncated n-terminal fragments lacking the YCKSCYG domain failed to bind the HTE-1 site. In addition, other EMSA studies have indicated that IL-10-induced tyrosine phosphorylation of the intact nt-nls peptide or the IL-10E1 protein promoted a 4–5 fold increase in binding activity compared to nonphosphorylated protein and recombinant protein from phagemid expression vectors (data not shown). The significance of these observations is currently under study in hopes of understanding how binding interactions of the YCKSCYG domain with specific nucleotide sequences of the HTE-1 element serve to regulate TIMP-1 expression.

Interestingly, the sequence data reported in this paper indicate that IL-10E1 is a ‘LIM-domain’ protein with a >95% homology to CRP2 and hsmlim genes (Weiskirchen *et al*, 1997). Sequence comparisons from GenBank indicated that there was 100% homology in the 5′ region spanning the first LIM domain of the CRP2 and hsmlim genes. However, the region spanning the second LIM-domain (∼ +451 to +490 bp)(i.e. exons 2–3) exhibited ∼90% homology with CRP2 and hsmlim genes (Weiskirchen *et al*, 1997). The deduced amino-acid sequence revealed that unlike CRP2 and hsmlim proteins (Dawid *et al*, 1995,^1998^; Weiskirchen *et al*, 1997), which expressed two LIM domains, the IL-10E1 protein consisted of a single LIM domain at the n-terminal region of the protein. Presumably, this difference between IL-10E1 and CRP2 or hsmlim might explain why IL-10E1 is overexpressed in prostate cancer cells and functions as an IL-10-responsive signal molecule.

In brief, the LIM motif is a ‘helix-loop-helix’ domain that was originally identified in three developmentally regulated transcription factors, Caenorhabditis *elegans* Lin-11, rat Isl-1, and *C. elegans* Mec-3 (Freyd *et al*, 1990; Karlsson *et al*, 1990), but has since been identified in a variety of proteins. The LIM domain consists of a 52 amino-acid sequence CX_2_CX_16–23_HX_2_ CX_2_CX_2_CX_16–21_CX_2–3_(C/H/D) motif, which typically has been associated with DNA-binding homeodomains and kinase domains (Freyd *et al*, 1990; Karlsson *et al*, 1990; Dawid *et al*, 1995,^1998^). The LIM domain has been recognised as a distinct protein motif within LIM homeodomain proteins by both genetic and biochemical methods (Freyd *et al*, 1990; Karlsson *et al*, 1990; Way and Chalfie, 1994). The LIM domain has been found to mediate ‘protein–protein’ interactions of cytoplasmic and nuclear proteins. NMR analysis of the solution structure of the c-terminal LIM domain of two of these proteins, CRP1 (Perez-Alvarado *et al*, 1994) and CRP2 (Konrat *et al*, 1997; Weiskirchen *et al*, 1997; Wadman *et al*, 1994), has shown that two zinc-fingers constitute a LIM domain, which represent two separated structural entities held together in a distinct configuration by hydrophobic interactions (Dawid *et al*, 1998). Moreover, the c-terminal LIM domain in CRP1 and CRP2 has been found to closely resemble the zinc-finger in the DNA-binding domain of GATA1, suggesting that the LIM domain might specifically bind DNA (Weiskirchen *et al*, 1995).

There is a potpourri of evidence indicating that LIM homeodomain proteins or LHX proteins function as transcription factors and genetic evidence has supported this idea (Dawid *et al*, 1998). Several studies have shown that LIM-homeo-domain proteins can activate the expression of specific genes (i.e. insulin) or direct cellular differentiation in cooperation with other nuclear factors (Perez-Alvarado *et al*, 1994). Other examples include, E47/Pan-1 and Lmx1.1 transcription factors, which have been shown to interact with LMO2, a LIM only protein, and with TAL1, LYL1, and LYL2 to regulate gene activation (Sadler *et al*, 1992; Warren *et al*, 1994; Larson *et al*, 1996; Johnson *et al*, 1997; Dawid *et al*, 1998). Similarly, the rat cysteine-rich intestinal protein (CRIP), the human cysteine-rich protein (CRP), and rhombotin-1 and -2 have been implicated as protein crosslinkers in transcription regulation (Dawid *et al*, 1998). The IL-10E1 protein belongs to the LIM only homeodomain family of proteins, which bind specific cognate DNA elements to regulate gene expression (Dawid *et al*, 1998). Part of the LIM domain, the YCKSCYG region, appears to directly mediate binding, but the entire LIM domain and the ‘helix–loop–helix’ conformation of the 52 a.a. LIM domain is probably important for specific binding to the TIMP-1 promoter and the stimulation of TIMP-1 expression.

In conclusion, we have shown for the first time that IL-10 signalling in prostate cell lines involves a signal pathway distinct from the STAT 1/STAT 3 pathway identified in white blood cell lines. The signal cascade activates a novel LIM only protein, termed IL-10E1, which is phosphorylated at two specific tyrosine groups, Y57/Y62, to activate signalling and binding to the TIMP-1 promoter. As a consequence, TIMP-1 expression is upregulated. Since TIMP-1 overexpression has been associated with the blockade of metastasis in human prostate tumour xenograph model studies (Stearns *et al*, 1999), we suggest that IL-10 may indirectly prevent tumour metastasis by this mechanism. IL-10 does not appear to alter IL-10E1 levels since ELISA measurements indicate that PC-3 ML cells express pictogram quantities (10–30 pg ml^−1^) independent of IL-10. Also, in NPTX-1532 cells which do not normally express detectable IL-10E1, IL-10 failed to induce expression.

The preliminary studies further indicate that the IL-10/IL-10R axis and activation of IL-10E1 stimulates the upregulation of TIMP-1 production in NPTX-1532 cells. Thus, the IL-10/IL-10R axis is of import in controlling tumour metastasis and the development of therapeutic approaches which target this pathway is ongoing in our lab. Recently, green fluorescent protein has been employed to tag regulatory proteins and analyse the mechanisms controlling nuclear translocation in real-time. For example, Hagting *et al* (1999) showed in real-time studies that the translocation of fluorescently tagged wild-type (wt) cyclin B1 was controlled by phosporylation. In these studies, we will utilise cells transfected with wt and mutant IL-10E1 proteins tagged with green fluorescent protein to identify (in real time) therapeutic agents that specifically block signalling in response to specific ligands (IL-10, IL-4, INF).
